# Broadband acoustic skin cloak based on spiral metasurfaces

**DOI:** 10.1038/s41598-017-11846-1

**Published:** 2017-09-14

**Authors:** Xu Wang, Dongxing Mao, Yong Li

**Affiliations:** 1 Institute of Acoustics, School of Physics Science and Engineering, Tongji University, Shanghai, 200092 People’s Republic of China; 2 Shanghai Key Laboratory of Special Artificial Microstructure Materials and Technology, School of Physics Science and Engineering, Tongji University, Shanghai, 200092 People’s Republic of China

## Abstract

A skin cloak based on the acoustic metasurface made of graded spiral units is proposed and numerically investigated. The presented skin cloak is an acoustical layer consisting of 80 subwavelength-sized unit cells, which provide precise local phase modulation and hence resort the disturbed sound filed in such a way to hide the object to acoustic wave. Numerical simulations show that the suggested skin cloak both work well under normal and small-angled incidences. By taking the advantage of the spiral-typed metasurface, the suggested skin cloak is rather thin with thickness in the order around 1/7 of the wavelength of target frequency, moreover, the intrinsic characteristics of modest dispersion ensure the skin cloak provides remarkable acoustic invisibility in a broad frequency ranging from 2500 Hz to 3600 Hz.

## Introduction

Cloaking usually refers to hiding an object from incoming waves by intentionally bending and twisting waves through an artificial shielding structure. The coordinate transformation and the development of metamaterials provide great flexibility on the cloaking design^1–6^. Instead of these relative bulky complete cloaks made up of strongly anisotropic materials, carpet cloak, which hides an object by restoring the disturbed sound field in such a way as if it was reflected from a ground plane, is proposed^7^. Recent development of metasurface^8–14^ paves the way for the realization of the carpet cloak with ultrathin thickness with respect to the interested wavelength. Hence such an idea is then numerically and experimentally verified^15–17^. Ni *et al*. created an optic metasurface tightly wrapped over an object to render it free from optical detection at several frequencies^18^. Similarly, the idea of carpet cloak has been introduced to acoustic context, by Helmholtz resonators^19^ and membrane-capped cavities^20, 21^. However, the property of high dispersion of these resonant-type units limits the resulting structures working in a narrow frequency range. In addition, the cloaking carpet of these works are slopes, indicating that the core concept of both^19, 20^ can be interpreted as compensating the distorted field from a certain tilt-angled slope by introducing a corresponding constant-phase gradient^8^ to its surface.

To this end, to provide a more general scheme for carpet cloaking design for an arbitrary-shaped object, a government equation is deduced, which links the incident and reflected sound to the information of the surface profile and the required local phase modulation. Moreover, to achieve the goal of broadband cloaking, spiral units, which are coiling-space based artificial structures, are adopted to fabricate the cloaking carpet. Our results show that their inherent characteristic of modest dispersion^22^ ensures the resulting carpet cloak (also noted as skin cloak) works in a wide range compared to that reported in the literature.

## Results

**Figure 1:**
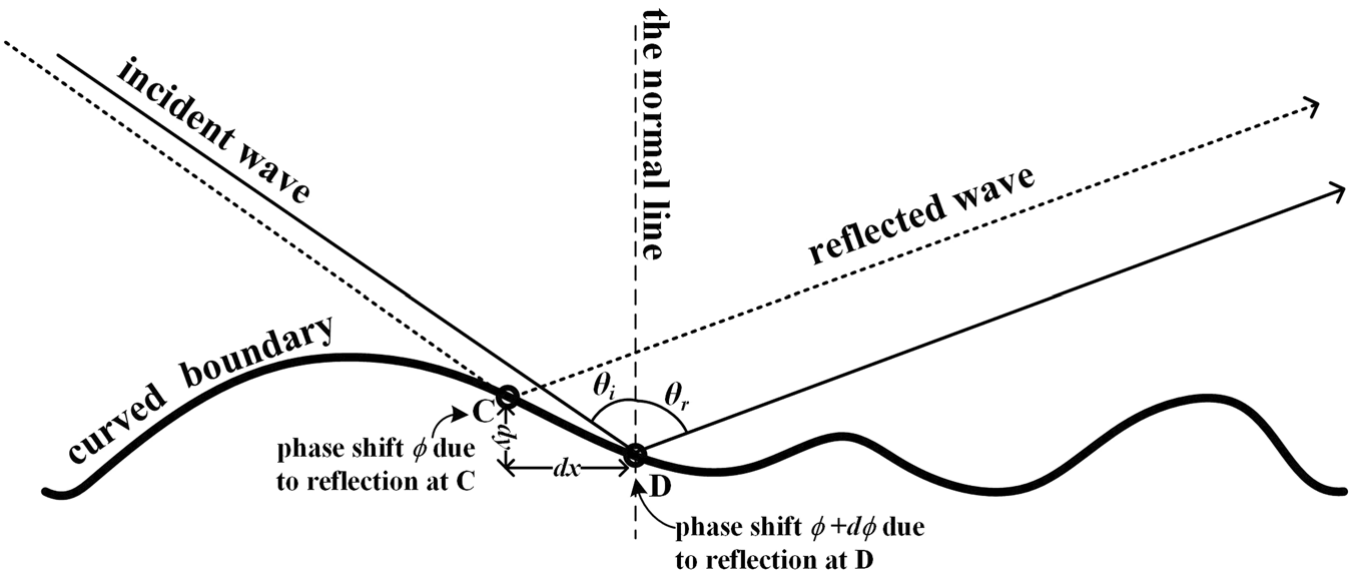
Reflection of a wavefront at an arbitrary-shaped object.

As illustrated by Fig. 1, when sound impinges on an arbitrary-shaped reflective object, the relation of the incident and reflected waves is given by

1 $$kdx({\rm{s}}{\rm{i}}{\rm{n}}{\theta }_{r}-{\rm{s}}{\rm{i}}{\rm{n}}{\theta }_{i})+kdy({\rm{c}}{\rm{o}}{\rm{s}}{\theta }_{r}+{\rm{c}}{\rm{o}}{\rm{s}}{\theta }_{i})=\frac{\partial \varphi }{\partial x}dx+\frac{\partial \varphi }{\partial y}dy,$$

where *k* is the wave number in the air, *θ*_*i*_ and *θ*_*r*_ represent the incident and reflected angles respectively, while the right part of the equation denotes the differential of the phase shifts between two nearby points on the object surface, i.e. (∂*ϕ*/∂*x*)*dx* + (∂*ϕ*/∂*y*)*dy*.

To cloak such an object means to modulate its reflected field in a way that mimics the reflected field from a ground. And this can be accomplished by covering the object with a metasurface having a distributed impedance in a subwavelength scale. Ideally, the metasurface is expected to provide accurate phase modulation (*dϕ* = 2*kdy* cos*θ*_*i*_) to compensate the distortion from the complex height profile of the object, indicating the relation between the incident and reflected angles when wave reflects on the metasurface can be expressed as

2 $$kdx(\sin \,{\theta }_{r}-\,\sin \,{\theta }_{i})+kdy(\cos \,{\theta }_{r}+\,\cos \,{\theta }_{i})=2kdy\,\cos \,{\theta }_{i}.$$

Obviously, Eq. 2 indicates that the reflected wave appears in the direction *θ*_*r*_ = *θ*_*i*_. However, in practice, such a metasurface is a locally reacting surface^7, 9–12, 15–20, 23, 24^, whose impedance is independent with the incident angle. Hence, for the carpet cloaking design, Eq. (1) can be rewritten as

3 $$kdx(\sin \,{\theta }_{r}-\,\sin \,{\theta }_{i})+kdy(\cos \,{\theta }_{r}+\,\cos \,{\theta }_{i})=2kdy,$$

which can be regarded as the approximation of Eq. (2), rendering the resulting reflected field from the covering metasurface mimics that from the ground. By linking the incident and reflected sound to the phase shift provided by local reacting impedance, Eq. (3) becomes the governing equation for our cloaking design for any object.

**Figure 2:**
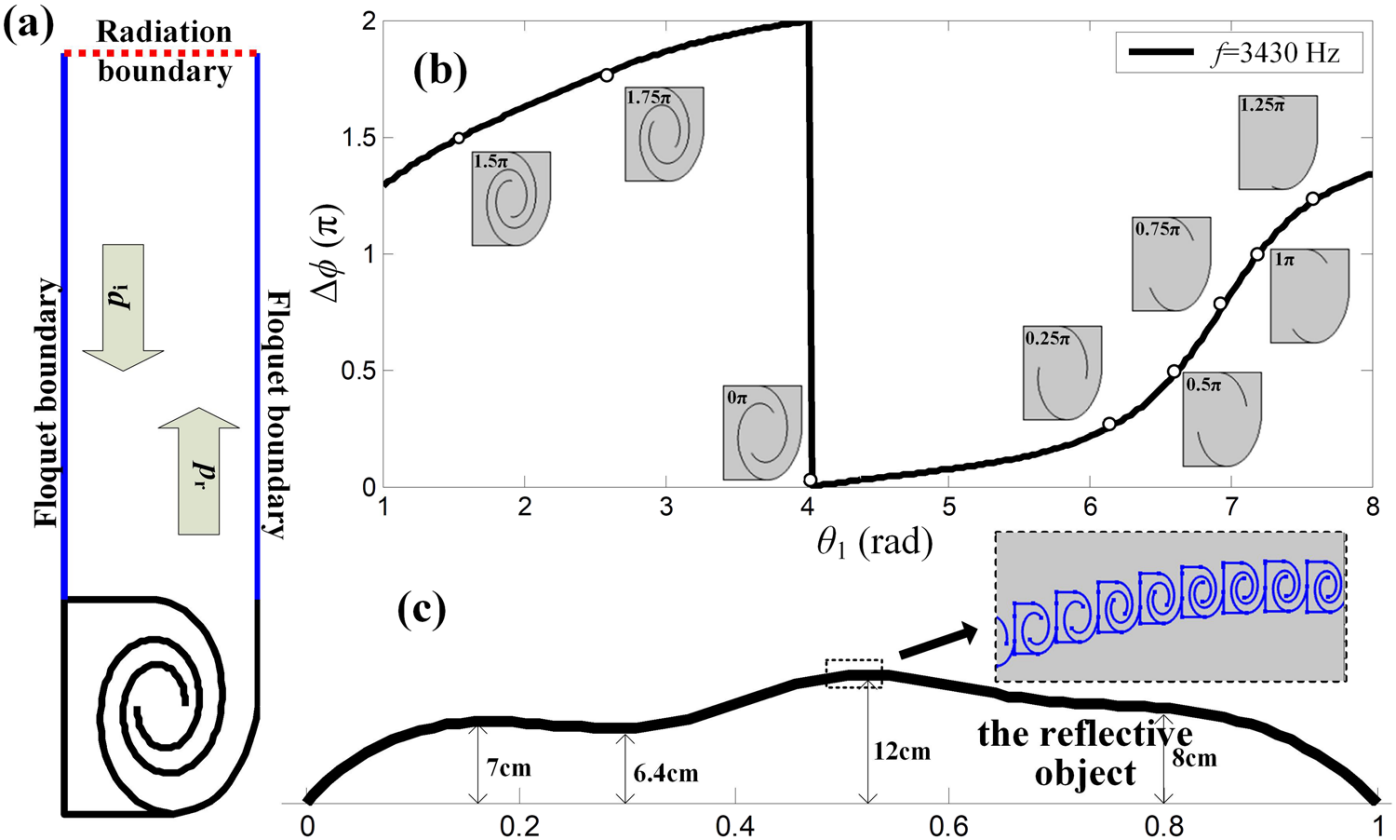
(**a**) Schematic diagram of the numerical simulation of the reflective coefficient of a spiral unit cell. (**b**) The variation of the phase shift provided by a spiral unit with its geometrical parameter *θ*_1_, the selected eight unit cells covering a full range of 2*π* with a step of *π*/4. (**c**) The height profile of a 2D object and the designed skin cloak cover on it, the inner figure zooms out the detail of the fabricated metasurface used for skin cloak.

The spiral structures, which are based on the concept of coiling up space, are selected as building blocks to fabricate the skin cloak. An original spiral structure is very similar to gastropod shells, whose geometries can be expressed in a parametric form as *r*(*θ*) = *ae* ^*bθ*^ (*θ*_1_ < *θ* < *θ*_2_)^22^. Here the angular span ranges from *θ*_1_ to *θ*_2_. Here, as shown in Fig. 2(a), a modified spiral unit is suggested by slightly changing its inner details and anisotropically scaling its thickness and width, which are set to 1.5 cm and 1.25 cm, respectively.

To retrieve the phase shift of the reflected sound contributed by the spiral unit, a full wave simulation by finite element modeling using COMSOL Multiphysics is performed. Figure 2(a) shows the schematic diagram of the simulation, which is a spiral unit cell mounted at the end of a waveguide. By sweeping the geometrical parameter *θ*_1_, the dependence of the phase shift Δ*ϕ* provided by the spiral unit to *θ*_1_ can be obtained, as illustrated in Fig. 2(b). According to this phase shift curve, eight unit cells, which are illustrated by the insets of Fig. 2(b), are chosen covering a 2*π* range with a step of *π*/4, and the values of these units are labeled with eight dots in Fig. 2(b). It should be noticed that such spiral units feature gradually changing channel width. For the eight selected units, even the width of the finest part of unit 1, i.e. the leftmost one in Fig. 2(b), is around 50 times higher than the thickness of the thermodynamic boundary layers (34 *μ*m at 2000 Hz—the lowest frequency of interest). Besides, our design does not introduce the resonant states of these units, since the resonance will significantly magnify the effect induced by viscosity. As a result, viscosity will not degrade the functionality of our design; and this has been confirmed by a similar work on coiling-space based metasurfaces^25^.

A two-dimensional reflective object with multiple bumps and dents is chosen, whose width is set to 1 m and height profile is mapped out in Fig. 2(c). When sound impinges on the object, the scattered wave is induced by the profile of its curved surface, resulting a disturbed reflected filed distinct from a reflective ground. To cloak such an object means to resort the wavefront scattered by the object. Thanks to the selected eight spiral units which offer a full 2*π* range phase shift at a step of *π*/4, this task can be accomplished by compensating the induced phase shift related to the local height of the surface by introducing a corresponding local phase modulation contributed by an appropriately configurated spiral unit. Here, these eight units shown in Fig. 2(b) are the building blocks used to assemble the metasurface, an array of 80 precise aligned spiral units, which is then tightly covered on the object. The details of the metasurface are zoomed out in the inset of Fig. 2(c). Given that the target wavelength *λ* is 0.1 m and the width of the object is 1 m, the width and thickness of our selected spiral units ensure the resulting metasurface possessing both a deep-subwavelength resolution and an ultrathin thickness.

**Figure 3:**
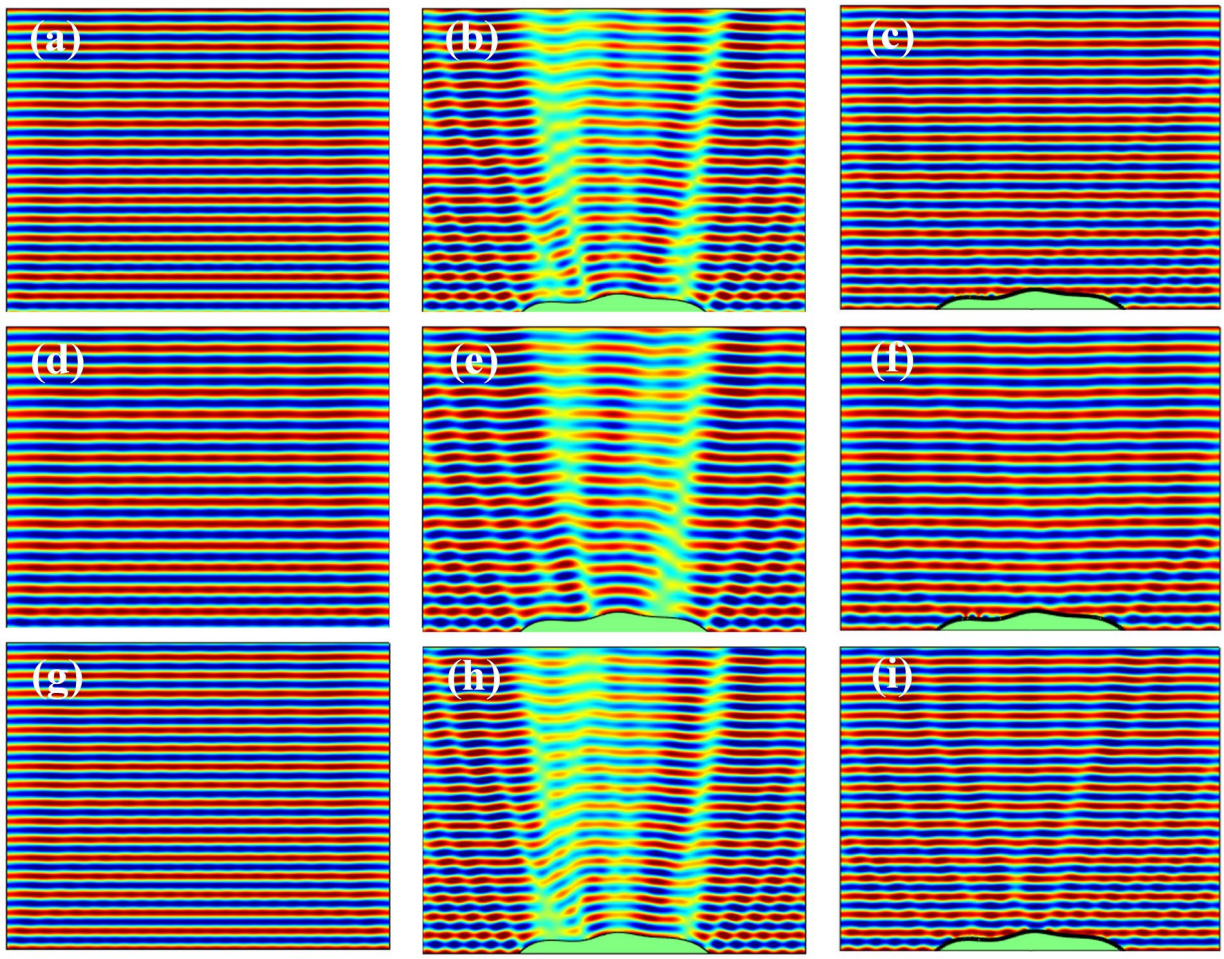
Simulated reflected sound fields of the reflective ground (left column), uncloaked object (middle) and the object covered by the metasurface (right) exposed to the normally impinging waves at 3430 Hz (upper row), 3000 Hz (middle) and 3600 Hz (bottom).

To verify the performance of the designed skin cloak, numerical simulations are carried out for the cases when a monochromatic wave impinges normally on the reflective ground, the uncloaked object, and the object covered by the metasurface. The reflected fields for these three cases at the target frequency *f* = 3430 Hz are given in Fig. 3(a–c). As expected, the sound field over the ground is significantly distorted by the presence of the object when no cloaking treatment is applied. Such a strong contrast between the object region and the surroundings implies that the object can be easily detected by scanning the disturbed field. In comparison, covering the object with the designed metasurface successfully restores the sound field, rendering the object completely hidden behind the cloaking skin. The slight discrepancies between the reflected fields of the cases with ground and the cloaked object may due to the discretization of the phase shift when building the metasurface.

Thanks to the characteristic of modest dispersion of the selected spiral units^22^, the capability of the suggested metasurface on hiding the object not only is remarkable at the target frequency, but also acceptable at nearby frequencies. Figure 3(d–f) show the comparison of the cases with ground, the uncloaked and cloaked object at 3000 Hz; while those at 3600 Hz are given in Fig. 3(g–i). At 3000 Hz, the designed metasurface behaves similarly to that at the target frequency by successfully resorting the disturbed filed; while at 3600 Hz, although a performance degradation can be observed, such a metasurface still provide acceptable invisibility compared to the uncloaked case.

**Figure 4:**
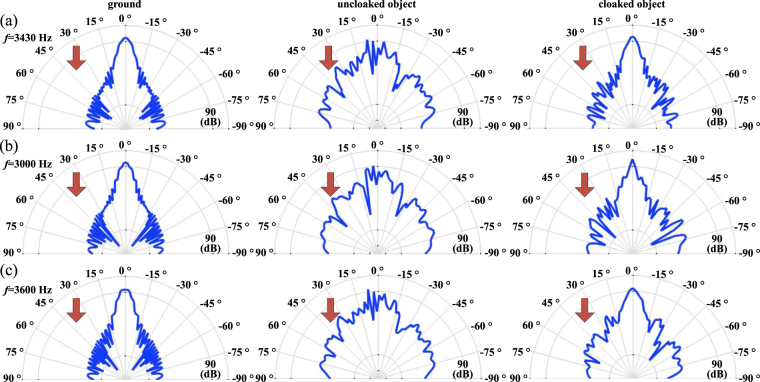
Scattering patterns of the ground (left column), uncloaked (middle) and cloaked (right) object under normal incidence at (**a**) 3430 Hz, (**b**) 3000 Hz and (**c**) 3600 Hz.

To further provide an intuitive image about the scattering features of our design, the scattering patterns of the ground, uncloaked and cloaked object described in polar coordinate are shown in Fig. 4. Such scattering features are numerically evaluated at the far field — 10 m (ten times higher than the object width) from the center of the object — to demonstrate the angular dependence of the scatter waves in sound pressure level. Figure 4(a) shows the scattering patterns at the target frequency (3430 Hz). Obviously, the suggested cloak provides a similar pattern with that above the ground. In contrast, the uncloaked object scatters the incident waves into all directions. Figure 4(b) shows the scattering patterns at 3000 Hz. Although a performance degradation can be observed, the suggested cloak provides a scattering field whose main lobe concentrating on the expected direction with its amplitude as least 25 dB higher than lobes in other directions (corresponding to the scattering energy at least 316 times higher than those in other directions). Similar results can be found in Fig. 4(c), which shows the patterns at 3600 Hz. It should be noted that the scattering patterns at these selected frequencies are just examples. Thanks to the intrinsic characteristics of the modest dispersion of the spiral units, such phenomena can be observed in a broadband ranging from 2500 Hz to 3600 Hz, within which the main lobe in the expected direction keeps 25 dB higher than other lobes. These results confirm the broadband characteristics of suggested skin cloak.

**Figure 5:**
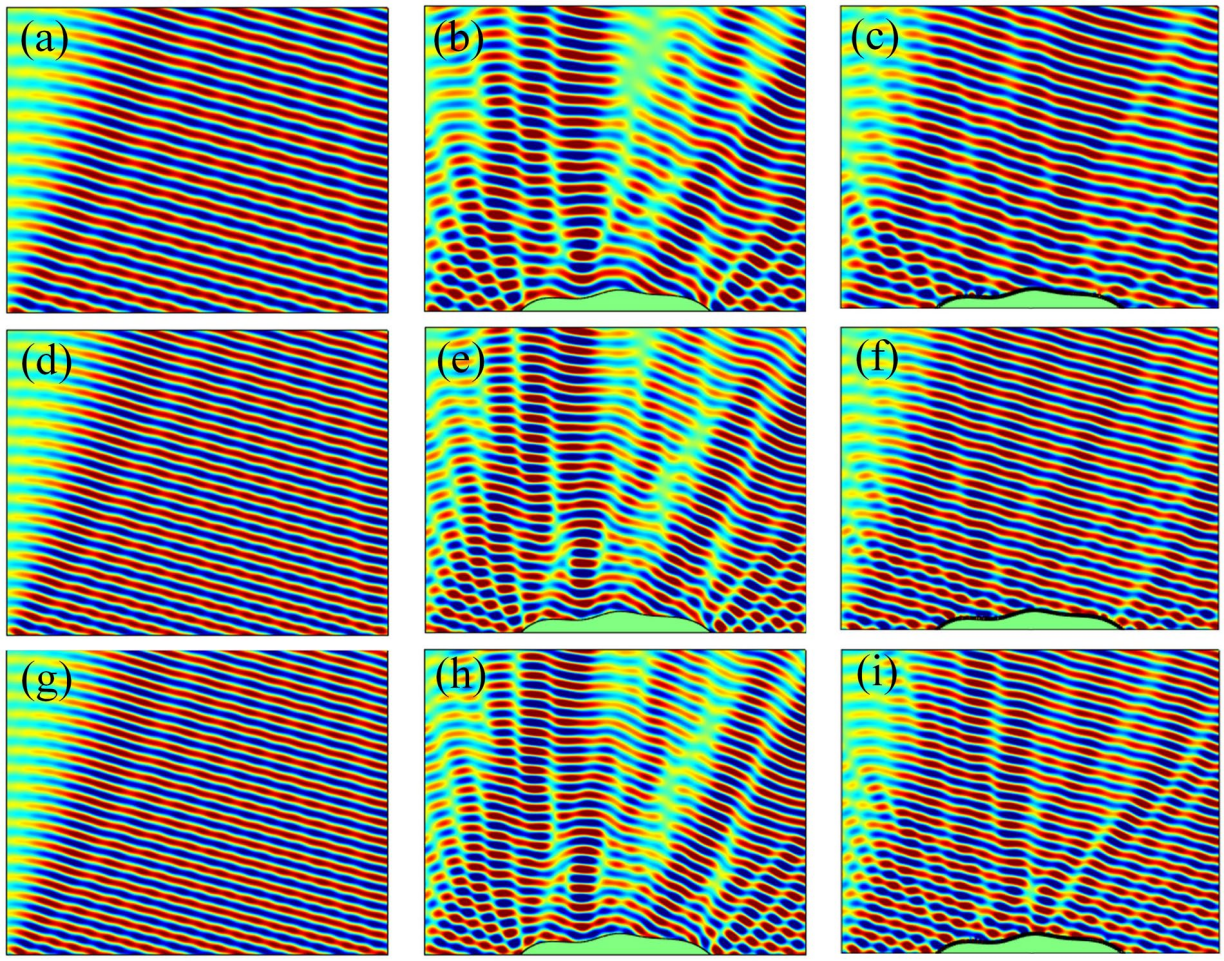
Simulated reflected sound fields of the reflective ground (left column), uncloaked object (middle) and the object covered with the metasurface (right) exposed to 15° impinging acoustic waves at target frequency 3430 Hz (upper row), 3000 Hz (middle) and 3600 Hz (bottom).

Although the proposed skin cloak is designed for the object exposed to normal wave incidence, observation on its governing equation, i.e. Eq. (3), suggests that the cloak may provide similar performance under small-angled incidence. This is further confirmed by the simulated results shown in Fig. 5, which gives the reflected sound fields above the ground and the object without and with the skin cloak when waves impinge obliquely from upper left with an angle of 15° to the normal of ground. Comparison on Figs 3 and 5 indicates that, when waves impinge obliquely, the performance of the suggested skin cloak, although poorer than that under normal incidence, is still remarkable in a wide frequency range. In principle, since the invisibility is achieved via local phase adjustments, the suggested skin cloak is expected to work well when waves coming from the other side (upper right).

**Figure 6:**
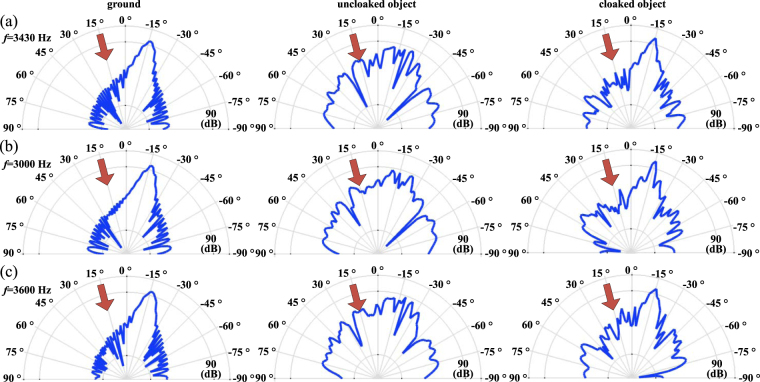
Scattering patterns of the ground (left column), uncloaked (middle) and cloaked (right) object at (**a**) 3430 Hz, (**b**) 3000 Hz and (**c**) 3600 Hz.

Figure 6 further provides the scattering patterns of the ground, uncloaked and cloaked object under small-angled incidence (*θ*_*i*_ = 15°). At the target frequency shown in Fig. 6(a), the suggested cloak successfully resorts the disturbed field by tailoring the scattering waves into the expected direction, providing a scattering pattern similar to that from the ground. As shown by Fig. 6(b) and (c), at 3000 Hz and 3600 Hz, the skin cloak provides a poorer yet somehow acceptable performance (main lobe in the expected direction is 18 dB higher than other lobes). Last but not least, for practical use, it is of interest to further consider the case with a larger incident angle. Actually, the results for *θ*_*i*_ = 15° imply that a growing *θ*_*i*_ results in a narrower working band. In general, our results show the significances of the suggested skin cloak, indicating that our design not only works in a broad frequency band, but also is applicable for a relative wide-angled detection.

## Discussion

In summary, a skin cloak based on the acoustic metasurface has been proposed and numerically validated. The designed skin cloak successfully hides an object to acoustic waves by restoring the disturbed reflected field. Since the acoustical invisibility is achieved by providing precise local phase adjustments, the suggested skin cloak has the potential to hide any object, even with sharp edges. Besides, adopting finer units (i.e. higher spatial resolution) may further enhance the performances of the cloak around the region with abruptly changing object profile. Moreover, given that the metasurface is assembled by the spiral units functioned as building blocks, the designed skin cloak fully takes advantage of the spiral-typed metasurfaces. It is rather thin yet provides remarkable cloaking in a broad frequency range. It is even competent for small-angled acoustical detection.

Notice that the presented skin cloak is just an example. By adopting the proposed design scheme, it should be fairly straightforward to conceal objects with different sizes by assembling different number of spiral building block. And it is flexible for different detecting frequencies by appropriately scaling the spiral units. All these remarkable advantages make the realization of practical skin cloak one step closer.

## Methods

The numerical simulations in this paper are performed by using the acoustic module of COMSOL Multiphysics, a commercial software package based on finite-element method. For the simulation on a single spiral unit, the inwalls of the unit cell are set to be acoustically rigid. In order to consider effects of adjacent units, the boundaries of the waveguide are set to Floquet boundary condition; while the upper boundary is assigned as plane wave radiation. For the simulations on spiral-unit constructed metasurface, the upper, left and right boundaries of the calculation domain is bounded by perfectly matched layers (PMLs), which are artificial absorbing layers allowing waves to propagate out from the domain without reflection. In all simulations, to ensure numerical accuracy, a fine mesh is used to ensure that the longest side length is kept below one tenth of the smallest wavelength of interest. Only the lossless case is considered for the time being. The target frequency is set to *f* = 3430 Hz, the medium is air with *c* = 343 m/s and *ρ* = 1.2 kg/m^3^.
